# Correction: Subjective and psychophysical olfactory and gustatory dysfunction among COVID-19 outpatients; short- and long-term results

**DOI:** 10.1371/journal.pone.0329646

**Published:** 2025-08-04

**Authors:** Mads Mose Jensen, Kasper Daugaard Larsen, Anne-Sophie Homøe, Anders Lykkemark Simonsen, Elisabeth Arndal, Anders Koch, Grethe Badsberg Samuelsen, Xiaohui Chen Nielsen, Tobias Todsen, Preben Homøe

In Table 3, the headings Subjective olfactory dysfunction and Psychophysical olfactory dysfunction are swapped in the second and third columns. The second column should be Psychophysical olfactory dysfunction and the third should be Subjective olfactory dysfunction. Please see the correct Table 3 here.

**Table 3:** Subjective and psychophysical test performance.

	Psychophysical olfactory dysfunction	Subjective olfactory dysfunction
Sensitivity	75.9%	58.6%
Specificity	78.6%	91.1%
Positive Predictive value	78.5%	87%

Sensitivity, specificity and positive predictive values of subjective and psychosocial olfactory dysfunction as diagnostic test for SARS-CoV-2 positivity

## References

[1] Jensen MM, Larsen KD, Homøe A-S, Simonsen AL, Arndal E, Koch A, et al. Subjective and psychophysical olfactory and gustatory dysfunction among COVID-19 outpatients; short- and long-term results. PLoS One. 2022;17(10):e0275518. doi: 10.1371/journal.pone.0275518 36191024 PMC9529127

