# Supplementary material for: Subjective and psychophysical olfactory and gustatory dysfunction among COVID-19 outpatients; short- and long-term results
Source: PLoS One. 2022 Oct 3;17(10):e0275518. doi: 10.1371/journal.pone.0275518 (PMC9529127; doi:10.1371/journal.pone.0275518)
Supplement: Attachment — Submitted filename: Revision doc.pdf [file pone.0275518.s001.pdf]

Congratulations to the authors for the study.

Below are some recommendations for the study.

Title

1) "Subjective and objective olfactory…" – No objective assessment was performed in this study.

Methods

Subjects

2) Line 73 – The sample included individuals aged between 18 and 80 years. However, it is known that the olfactory function, as in other sensory systems is impaired with aging. Therefore, it is very common for individuals after the age of 60 to present alterations in their sense of smell. The sample should have a lower age range, perhaps between 18 and 50 years, due to aging. In addition, these individuals did not undergo a smell assessment before being infected with COVID-19. What if, before the infection, they already had a slight or moderate change in the identification of odors, which they had not noticed? Therefore, it is important to review the age group.

3) Line 86 – I suggest removing "Objective tests" and putting "subjective tests". See below.

4) Lines 95 and 96 – The authors state that the taste test - Burghart's Taste Stips and the olfactory test - Brief Smell 97 Identification Test are objective tests that were performed by an otolaryngologist. However, these tests are not objective, but subjective/psychophysical, as they depend on the individual's response. The only objective olfactory test is the event-related Olfactory Evoked Potential and the Electroolfactogram, which were not performed in the present sample.

5) Line 95 – How are these tests evaluated for normality and degrees of olfactory and gustatory loss? There is no description for the reader throughout the article.

6) Line 99 – No objective, gustatory or olfactory test was performed in this study. I recommend removing that phrase.

7) Line 107 – The authors state that a questionnaire was made to obtain demographic data and questions 1-6 of the SNOT-22 questionnaire were added. Why didn't you complete the SNOT-22 questionnaire, as it is validated and used internationally? It would be interesting and important to have carried out a complete assessment of the quality of life of each participant during the study period.

8) Line 109 – All participants answered this modified questionnaire four times during the study: at baseline, 30, 60, and 90 days? Is it possible to trust that these questionnaires were actually answered at these times since they were sent by e-mail? Were the psychophysical tests redone 30, 60, and 90 days after the first assessment? Or at least at the end of the study? This is not clear from the text.

Statistical methods

9) Line 112 – There are no objective methods for the assessment of smell and taste in this study.

10) Line 113 – The BSIT test is not objective, it is psychophysical.

Results

11) Lines 122 to 127 – I suggest putting this data in a table and drawing attention in writing only to the most important result.

12) Line 128 – The authors have already put in the title of the table what is being exposed here. I recommend removing and adding the caption.

13) In table 1 – it is necessary to place a legend below the table, for the acronyms PCR, OD, and GD and for other information that is necessary for the understanding of the table.

14) In table 1, in the “Reported symptoms and risk factors of OD/GD” part, add the symbol of (n) for the total number of participants and (%) to identify that the data are being presented in percentage.

15) In table 1, in the “Objective assessment” part, I suggest putting “ENT assessment” and also indicating that the data are presented as a percentage.

16) Line 134 – The acronym IQR is cited for the first time, but there is no full description of - Inter Quartile Range. This description will only occur on line 154.

Olfactory dysfunction

17) Lines 131 to 141 – There is no need to write down all the data that is already presented in the table. Comment only the most important result and tell the reader to analyze the data table.

18) Lines 142 and 143 – Table 2: This information would be better as a table title. I suggest improving the title and adding the legend below the table for the acronyms and other information that is necessary for the understanding of the table.

19) Lines 145 to 152 – the results described are in which table or graph? Please put the reference.

20) Lines 151 and 152: Please, make a table with the results of diagnostic tests for sensitivity, specificity, and positive predictive value.

21) Again, I recommend focusing only on the most important result and not writing out all the results that are already in the tables or graphs.

22) Line 157 (Figure 1) - I recommend improving the quality of the figure.

23) Line 161 - remove 'table 3' and leave only the explanation about the table and add the necessary captions.

Gustatory Dysfunction

24) Lines 167 to 175 – I recommend bringing the results of gustatory dysfunction together with the olfactory one so that the reader has table 2 just below for analysis. Do not describe the results already presented in the table in full. And don't forget to indicate the table.

25) Line 176 (Figure 2) - I recommend improving the quality of the figure**.**

Discussion

26) Line 181 – Replace "objective tests" with "psychophysical tests".

27) Lines 181 to 185 – It is interesting to start the discussion by stating the main and most important result of the study. The description presented in this paragraph has already been done in the methodology.

28) Line 194 – "(…) suggesting that some individuals fail to recognize their OD." This is important to point out, as many individuals cannot have a clear perception of how the sense of smell is.

29) In the discussion also review the description of what are subjective and objective tests, because as already mentioned above, all tests performed by the doctor were psychophysical and not objective. The previous questionnaire applied for the selection of participants is a self-report of olfaction.

30) Do not put so many results, as these are already in the tables. Discuss them only and focus only on the most important ones. Discussion is the crucial part to discuss the study data with what there is already literature.

Limitations

31) The authors report important limitations, mainly the bias in filling out the initial questionnaire.

Conclusion

32) Do not put statistical data in the conclusion. And do not summarize the study in this part.

33) The conclusion should be brief and answer your research question/objective. Do not put information that has already been discussed in the article or that is in the methodology.

34) Objective: "This study aimed to examine subjective and objective olfactory and gustatory function in non hospitalized individuals with acute COVID-19 up to 6 months

after infection." Answer in the conclusion: Was there an improvement in olfactory and gustatory function, after 6 months of the initial diagnosis of COVID?

35) Recommendation: send the article to be reviewed by a native speaker of English.
